# Large muscles are beneficial but not required for improving thermogenic capacity in small birds

**DOI:** 10.1038/s41598-018-32041-w

**Published:** 2018-09-18

**Authors:** Myriam S. Milbergue, Pierre U. Blier, François Vézina

**Affiliations:** 1 Département de biologie, chimie et géographie, Université du Québec à Rimouski, Rimouski, Québec Canada; 2 Groupe de recherche sur les environnements nordiques BORÉAS, Rimouski, Québec Canada; 3 Centre d’études nordiques, Rimouski, Québec Canada; 4 Centre de la Science de la Biodiversité du Québec, Rimouski, Québec Canada

## Abstract

It is generally assumed that small birds improve their shivering heat production capacity by developing the size of their pectoralis muscles. However, some studies have reported an enhancement of thermogenic capacity in the absence of muscle mass variation between seasons or thermal treatments. We tested the hypothesis that an increase in muscle mass is not a prerequisite for improving avian thermogenic capacity. We measured basal (BMR) and summit (M_sum_) metabolic rates of black capped chickadees (*Poecile atricapillus*) acclimated to thermoneutral (27 °C) and cold (−10 °C) temperatures and obtained body composition data from dissections. Cold acclimated birds consumed 44% more food, and had 5% and 20% higher BMR and M_sum_, respectively, compared to individuals kept at thermoneutrality. However, lean dry pectoralis and total muscle mass did not differ between treatments, confirming that the improvement of thermogenic capacity did not require an increase in skeletal muscle mass. Nevertheless, within temperature treatments, M_sum_ was positively correlated with the mass of all measured muscles, including the pectoralis. Therefore, for a given acclimation temperature individuals with large muscles do benefit from muscle size in term of heat production but improving thermogenic capacity during cold acclimation likely requires an upregulation of cell functions.

## Introduction

For small avian species wintering at high latitudes, winter acclimatization is mainly a physiological phenomenon^1–5^ where cold hardiness is improved as temperature decline from fall to peak of winter^5–8^. This improved capacity is typically associated with increases in basal (BMR) and summit (M_sum_) metabolic rates, which are respectively thought to reflect physiological maintenance costs^9–13^ and cold endurance^4,12,14,15^.

The seasonal elevation in BMR is often interpreted as resulting from an increase in daily food consumption requiring larger digestive and excretory organs (e.g. liver, gizzard, intestine), in turn leading to higher maintenance cost^7,16–19^. However, as the influence of body composition on BMR is context-specific^20,21^ and can be affected by tissue metabolic intensity^10,22–25^, this scenario may not be generalizable^13^. For example, in cases where acclimatization also leads to considerable increases in skeletal muscle size, the influence of digestive and excretory organs on BMR can be overshadowed by the amount of muscle tissues consuming energy during measurements^25,26^. In contrast, since M_sum_ is a measure of maximal shivering heat production^2,27,28^, the influence of skeletal muscle size, particularly the flight muscles and heart size, on thermogenic capacity appears much more consistent. In several small free-living wintering species, elevated winter M_sum_ is indeed associated with seasonally larger pectoralis muscles^5,26,29–33^. The mass of skeletal muscles and heart has also been found to correlate significantly and positively with M_sum_ several times^19,25,26,34–37^.

Despite the seasonal changes in muscles size and M_sum_ observed in the wild, a small number of studies, although they were not designed to investigate this specific phenomenon, reported improvements of thermogenic capacity in controlled conditions independently from changes in muscles size^19,38,39^. This phenomenon was found to occur even in species known to increase pectoralis muscle mass in winter. For example, dark-eyed juncos (*Junco hyemalis*) are known to increase both their M_sum_ and the size of their pectoralis muscles in winter relative to summer^29^ but recently, Swanson *et al*. [ref.^38^, see also^40^] conducted an experiment with captive juncos and found a 16–19% higher M_sum_ in cold-acclimated (3 °C) birds relative to individuals exposed to a warm treatment (24 °C) with no difference in pectoralis muscle mass. Similarly, Barceló *et al*.^19^ documented a 19% higher M_sum_ in captive white-throated sparrows (*Zonotrichia albicollis*) acclimated to −8 °C compared to individuals maintained at thermoneutrality (28 °C). In this particular case, although the expected positive correlation between heart and muscles mass and M_sum_ was found in cold acclimated birds, there was no significant difference in the mass of pectoralis or other skeletal muscles between thermal treatments.

Since shivering does not produce external work, and thus most of the chemical energy consumed during contraction is released as heat^40^, there is no doubt that large muscles should produce more heat, for a given level of shivering, compared to small muscles. Consequently, in a given dataset if muscles mass and M_sum_ cover a range of variation sufficiently wide, which is often the case with interseasonal studies^5,26,30,31,35^, positive correlations between muscle mass and M_sum_ should be detectable across or within seasons. However, the experimental evidence presented above suggest that developing larger muscles may not be an obligate prerequisite for improving individual thermogenic capacity, even in species known to increase the size of their muscles during cold winters.

To test this hypothesis, we conducted an experimental study with captive black-capped chickadees (*P. atricapillus*). Chickadees are small (11 g) non-migratory passerines that typically express elevated M_sum_ in winter relative to summer^8,26,41^. This improvement of thermogenic capacity parallels or statistically correlates with the development of larger muscles and heart^26,31,42^. However, at least one case of chickadees going through a cold winter without significant changes in pectoralis muscles size has been documented^43^. This suggests that in experimental conditions, where only temperature is manipulated, this species could also show improvement of thermogenic capacity independently from skeletal muscle mass variation. We therefore exposed birds to two thermal treatments (−10 °C and 27 °C), with the expectation that cold acclimated birds, would show a higher M_sum_ than those maintained at thermoneutrality but no difference in mean mass of pectoralis and other skeletal muscles. We nevertheless expected a correlation between muscle mass and M_sum_ across or within treatments as individuals with larger muscles could still benefit from the mass of these tissues in terms of maximal shivering heat production, independently from their acclimation temperature. We also measured BMR in these birds to document maintenance costs. In this particular case, we expected that BMR variation across treatments would correlate with the mass of digestive and excretory organs if there was no major difference in muscle mass between treatments^19^. In contrast, we expected BMR to correlate with the mass of skeletal muscles if cold acclimated birds enlarged the size of these organs^26^.

## Material and Methods

### Birds collection and acclimation

From January to April 2015, we captured 49 black capped chickadees using mist nets at two sites, the Forêt d’Enseignement et de Recherche Macpès, (48°19N, 68°30W) and lac à l’Anguille (48°25N, 68°25W), both in eastern Québec, Canada. These birds were brought into captivity at the avian facilities of the Université du Québec à Rimouski. Birds were held in individual cages (39 × 43 × 31 cm) and exposed to a constant photoperiod (10 L:14D) for the remainder of the experiment. Birds consumed a diet of living mealworms and freshly-thawed crickets (0.20 g and 0.30 g per day, respectively), sunflower seeds, Mazuri small birds maintenance diet (MAZURI® exotic animal nutrition, USA) and water, which were available *ad libitum*. The birds also received vitamin supplements, daily (Electrolytes plus, Vetoquinol N.-A.INC, QC, Canada) and once per week (Poly-tonine A® complex, Vetoquinol N.-A.INC, QC, Canada) in their water. Our experimental groups were formed of 24 individuals maintained at −10 °C (cold) and 25 individuals maintained at 27 °C (thermoneutral zone of this species^41,44^). Birds were acclimated to these conditions for a minimum of 39 days (mean = 61.5, max = 84) after which we measured average daily food consumption over 6 days (same diet but excluding Mazuri) by subtracting the mass of food left in food trays in the morning from what had been offered the day before at the same time. Following the 6 days of food intake measurements, we proceeded with metabolic rate trials (see below).

All bird manipulations have respected the Canadian Council on Animal Care (CCAC) guidelines and were approved by the animal care committee of the Université du Québec à Rimouski (CPA-60-15-160). They also have been conducted under scientific and banding permits from Environment Canada–Canadian Wildlife Service.

### BMR and M_sum_ measurement

Because tissues collected on birds were also analyzed in another experiment that required fresh samples (results not shown, Milbergue *et al*., in prep), our measurements sequence for metabolic rate was limited to recording BMR and M_sum_ on 8 birds per week, until all birds had been measured (49 days). Each day of respirometry trial involved measurement on four birds (2 from each treatment) and followed the protocols described in details by Lewden *et al*.^45^ and Petit *et al*.^8,26^ where the animals VO_2_ were measured using FoxBox oxygen analyzers (Sable Systems, Las Vegas, NV, USA). Each M_sum_ trials were conducted on two randomly chosen birds from a same temperature treatment and began approximately at 9:00 and at 12:30 (alternating treatments between measures). Trials began by weighing the birds (0.00 g, Scout Pro, Ohaus, NJ, USA) and placing each of them individually in a stainless steel metabolic chamber (volume = 1350 ml). The birds then received air during 20 min before being exposed to helox gas (21% oxygen, 79% helium) using a flow rate of 900 ml min^−1^ controlled by mass flow controllers (Omega, FMA 5400/5500, QC, Canada) calibrated with a Bubble-O-Meter (Dublin, OH, USA). We used a sliding cold exposure protocol^46^, where ambient temperature was first set to either 0 °C (cold group) or 10 °C (thermoneutral group) and then ramped down by 3 °C every 20 min. Trials ended when birds became hypothermic, which was easily identifiable in real time as a steady decline in oxygen consumption for several minutes. Body temperature was immediately measured after taking birds out of their chamber using a thermocouple reader (NIST-traceable Omega model HH-25KC, QC, Canada) and a copper constantan thermocouple inserted into the cloacae, approximately 10 mm deep. Only data from birds showing a body temperature after trials lower or equal to 38.5 °C^47,48^ were used in the analyses. This removed five M_sum_ measurements from our sample. Body mass was again recorded at the end of trial and average body mass was used in statistical analyses on M_sum_.

After M_sum_, birds were brought back to their cage and had access to food and water until BMR measurement, starting at around 19:00. BMR trials were done on all 4 birds at 30 °C (thermoneutral zone^41,44^) in chambers that received 500 ml min^−1^ of dry, CO_2_ free air. Trials ended the following morning (at approximately 7:30). As for M_sum_, body mass was measured prior to and after BMR measurement and the average was used in statistical analyses. Birds were then returned to their cage.

Metabolic rates were calculated with the EXPEDATA software, v1.8.4 (Sable Systems, Las Vegas, NV, USA) using the equation 10.1 of Lighton^49^. M_sum_ and BMR were calculated from the highest and lowest averaged 10 min of VO_2_. Because birds use lipids as metabolic fuel during shivering^50^ and the duration of BMR trials (>720 min) insured that birds were post-absorptive at time of BMR measurement, we estimated heat production in Watts assuming an energy equivalent for lipid oxidation of 19.8 kJ l^−1^ O_2_^51^. Five individuals died of unknown cause during the experiment, leaving a final sample size of n = 20 (−10 °C) and 18 (27 °C) for BMR and n = 18 (−10 °C) and 19 (27 °C) for M_sum_.

### Organ collection

Birds were euthanized by decapitation in the 2 to 5 days following their respirometry trial (delay caused by measurements conducted on tissues in parallel to this experiment, Milbergue *et al*., in prep). The right and left pectoralis muscles, heart, liver, empty intestine, pancreas and gizzard were removed within minutes of the birds death and weighed (0.0001 g) with a precision balance (Cole-Parmer Symmetry, PA-Series, Canada). These organs were placed in Eppendorf tubes and immersed in liquid nitrogen before being transferred to a −80 °C freezer. Carcasses were preserved at −20 °C until we completed dissections. This was done by removing and weighing the brain, lungs, kidneys, skin (feathers removed) and upper right and left leg muscles, considered as a single organ and including bones. The remaining carcasses were therefore composed mainly of skeletal muscles and bones. Organs were then freeze-dried (FreeZone 2.5, Labconco, Kansas city, KS, USA) for 2 days to obtain constant dry mass of tissues^26^. Adipose tissues have low metabolic activity and can bias analyses on relationships between mass or body composition and metabolic rates when birds contain differing amounts of fat^52,53^. We therefore extracted lipids from these samples with a Soxhlet apparatus using petroleum ether to obtain final lean dry mass of organs.

It should be noted here that we do not have lean dry mass data for the heart as the entire organ was needed for tissue analyses (Milbergue *et al*., in prep). Wet mass is therefore presented for this organ. For pectoralis muscles and liver, since we used subsamples for tissue analyses and processed the remaining tissue as the other organs (freeze-drying and fat extraction), we recalculated lean dry mass of these organs in proportion to the original wet mass of the complete organ. In this experiment, we originally planned on obtaining ash-free lean dry mass for leg muscles and the remaining carcass but a technical problem during the burning of samples in a furnace led to the loss of a large number of samples. We therefore cannot present ash-free data.

### Statistical analysis

Our analyses first tested whether birds differed between treatments before the temperature change. We thus used one-way ANOVAs to test for a treatment effect (cold or thermoneutral) on furcular fat score (estimated according to Gosler^54^), structural body size and body mass measured prior to group formation. Structural body size was calculated as the first principle component (PC) from a principal component analysis combining variation in length measurements of head plus beak, tarsus, wing and tail^55–57^.

To determine how thermal environments might have influenced body composition after acclimation, we ran ANCOVA models testing for the effect of thermal treatment on organ lean dry mass. Since structurally larger birds might also have larger organs, we included body size as a covariate in these models. We used the same approach to determine the influence of thermal treatments on BMR and M_sum_. Models included the effect of time since capture to consider a potential influence of captivity duration, but this last variable was not significant and is therefore not considered further. The models were first run on whole BMR and M_sum_. We then included structural body size or body mass as covariate but the size effect was not significant in any models thus this effect is not presented here.

To determine the influence of body composition on metabolic performance, analyses are typically based on stepwise regressions or a model selection approach where the influence of all body constituents on BMR and M_sum_ are compared and ranked in order of significance and importance of their effect (e.g.^25,58^). However, results from these analyses depend on the variables included in models and missing variables can influence results^59^. In the present case, we could not include lean dry heart mass in our analyses. However, although this organ typically represents only 1% of total body mass, the heart has been shown to significantly contribute to variation in both BMR^25,60^ and M_sum_^19,25,26^. We therefore chose a simpler approach for our analyses. We conducted separate ANCOVA models including thermal treatment, lean dry mass of the organ (fresh mass for heart) and their interaction. These models were then ranked according to the Bayesian information criterion (BIC).

Analyses testing for the effect of muscle tissues on metabolic rates were first conducted considering muscle groups separately. Models thus included either pectoralis muscles, leg muscles (including bones) or carcass (including bones) as independent variables. As leg muscles and carcass mass included bones and bone mass should closely correlate with structural body size, we also included body size as an additional covariate in these models to control for bone mass. Then, we combined pectoralis muscles, legs muscles and carcass to generate a “total muscle” variable and used total muscle as our independent variable in the model. Here again, structural size was included as a covariate to control for bones mass. In all of these cases, however, the effect of structural body size was never found to be significant, likely because bones mass (measured as ash) only represents a small proportion of lean dry body mass in chickadees (27% of carcass mass, including all bones, based on data from^26^). This effect is therefore not included in the models presented here.

Analyses were conducted using R Studio (3.3.1) and JMP Pro (12.0.1). In all analyses, we eliminated non-significant interactions and variables to obtain final models. Normality of model residuals was confirmed in using Shapiro-Wilk tests and we used Cook distance test to identify outlier values and remove them from analyses.

## Results

### Treatment effect on body composition

Birds from both groups did not differ prior to treatment. There was no significant effect of treatment on furcular fat score (F_1,47_ = 2.4, P = 0.1), structural body size (F_1,46_ = 1.2, P = 0.3) or body mass (F_1,47_ = 0.97, P = 0.3). Groups did not differ in sex ratio either (χ² = 0.19, P = 0.66, sex determined during dissection). After 28 days of acclimation, differences were detected (Table 1). At the end of acclimation, cold-acclimated birds were eating 43.5% more food but nevertheless had, at the time of dissection, 27–30% less fat than individuals maintained at thermoneutrality (Table 1). Despite this difference in fat content, post-acclimation body mass did not differ significantly between treatments (Table 1). Among organs, only the heart (fresh mass), lungs, pancreas and leg muscles differed between experimental temperatures (Table 1). Cold-acclimated birds had a 10.7% and a 8.0% smaller lungs and legs muscles but had a 14.3% and a 95.8% larger heart and pancreas, respectively. No other organs differed between treatments, including pectoralis muscles, carcass, digestive and excretory organs. Total lean dry muscles mass differed by less than 1% between treatments (Table 1).

**Table 1:** Least square means (±s.e.m) and differences between cold (−10 °C: C) and thermoneutral (27 °C: T) treatments for body composition variables in black-capped chickadees.

Variable	Cold	Thermoneutral	F (df)	P	% difference (C relative to T)
Food intake	3.86 ± 0.09	2.69 ± 0.08	96.4 (1,45)	<0.0001	43.5
Mass and fat					
Body mass	11.96 ± 0.16	12.25 ± 0.16	1.5 (1,47)	0.2	−2.4
Total organ fat mass	0.88 ± 0.07	1.25 ± 0.06	16.1 (1,35)	<0.001	−29.6
Furcular fat mass	0.11 ± 0.01	0.15 ± 0.01	5.7 (1,35)	<0.05	−26.7
Muscles					
LD pectoralis	0.41 ± 0.01	0.39 ± 0.01	0.97 (1,34)	0.33	5.1
LD legs^a^	0.23 ± 0.005	0.25 ± 0.004	9.2 (1,33)	<0.01	−8.0
LD carcass^a^	1.21 ± 0.02	1.24 ± 0.02	0.83 (1,33)	0.37	2.4
LD total muscles^a^	1.86 ± 0.04	1.85 ± 0.04	0.0005 (1,33)	0.98	0.5
Cardio pulmonary					
Heart	0.16 ± 0.004	0.14 ± 0.004	12.0 (1,34)	<0.001	14.3
LD lungs	0.025 ± 0.001	0.028 ± 0.001	6.4 (1,35)	<0.05	−10.7
Digestive and excretory					
LD gizzard	0.087 ± 0.005	0.079 ± 0.005	1.35 (1,35)	0.25	10.1
LD intestine	0.039 ± 0.002	0.036 ± 0.002	1.6 (1,34)	0.22	8.3
LD liver	0.078 ± 0.004	0.066 ± 0.004	2.0 (1,25)	0.17	18.2
LD pancreas	0.0094 ± 0.0007	0.0048 ± 0.0007	23.6 (1,32)	<0.0001	95.8
LD kidneys	0.030 ± 0.001	0.029 ± 0.001	0.21 (1,35)	0.65	3.4
Other					
LD skin^a^	0.11 ± 0.003	0.11 ± 0.003	0.01 (1,33)	0.91	0.0
LD brain	0.10 ± 0.001	0.11 ± 0.001	0.78 (1,35)	0.38	−9.1
Total LD body mass^a^	2.33 ± 0.05	2.30 ± 0.04	0.18 (1,33)	0.67	1.3

Units are in grams except for food intake (g/day).

^a^Includes bone mass and controls for structural body size (see text for details).

### Influence of temperature and body composition on metabolic performance

**Figure 1:**
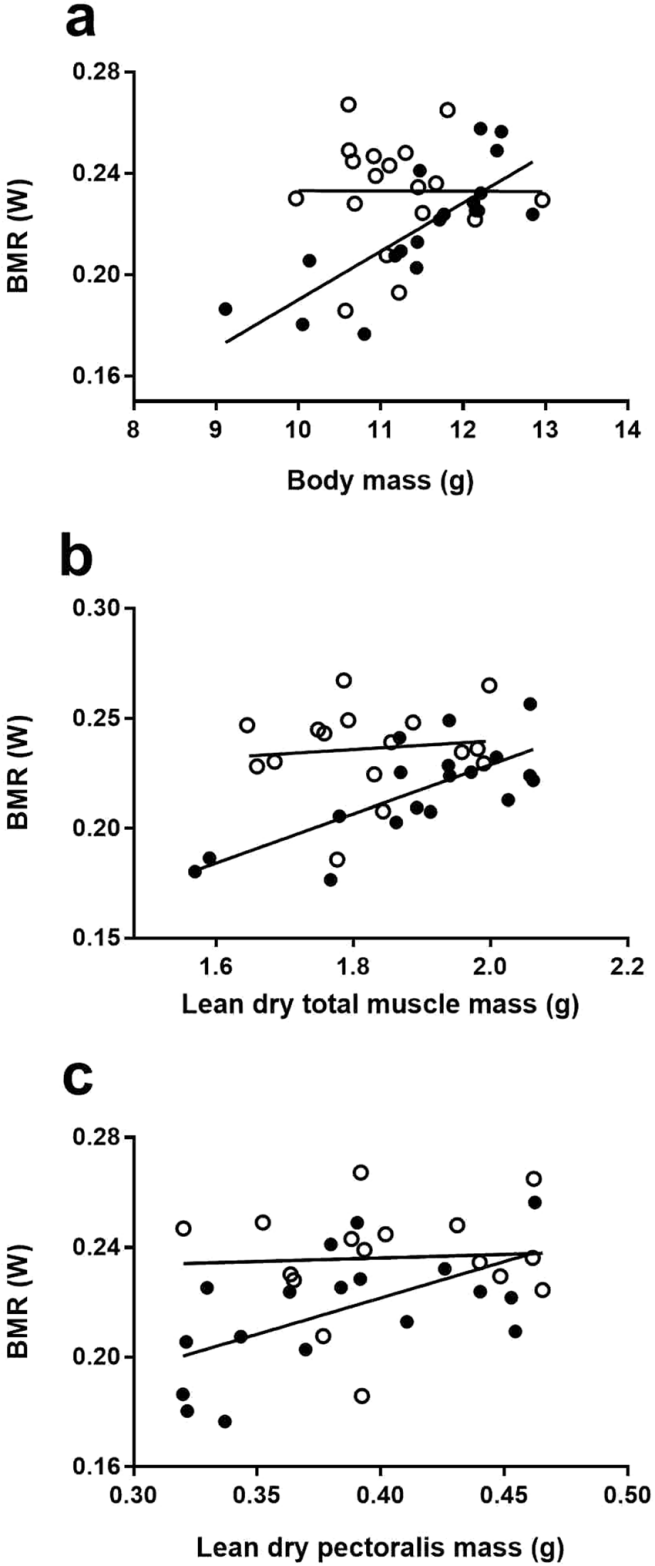
Relationships between BMR and body mass or lean dry mass of skeletal muscles in black-capped chickadees: (**a**) body mass, (**b**) total lean dry muscle mass (including bones), (**c**) lean dry pectoralis muscle mass (filled circles: 27 °C, open circles: −10 °C).

Cold-acclimated birds had a BMR 4.5% higher on average than individuals kept at thermoneutrality, but the temperature effect depended on body mass (interaction body mass*treatment, Table 2). Indeed, although the influence of body mass on BMR was clear at thermoneutrality (independent regression R^2^ = 0.61, n = 19, P < 0.0001), this effect appeared uncoupled at −10 °C (independent regression: P = 0.99, Fig. 1a). Therefore, most birds kept in the cold had a BMR as high as the heaviest birds kept at thermoneutrality (Fig. 1a). Ranking independent ANCOVA models for relationship between organ mass and BMR revealed a clear influence of skeletal muscle mass on BMR (Table 3). The model including total muscle mass (Fig. 1b) ranked first followed by the models including skin, carcass, leg and pectoralis muscle (Fig. 1c). Digestive, cardio-pulmonary and excretory organs all ranked after these organs and thus apparently had less influence on BMR variation (Table 2).

**Table 2:** Effects of thermal treatments, body mass on BMR and M_sum_ and least square means (±s.e.m) per treatment.

	Treatment		Body mass		Interaction		Cold	Thermoneutral	% difference
	F (df)	P	F (df)	P	F (df)	P	(Watts)	(Watts)	
BMR	7.15 (1,33)	<0.05	0.0002 (1,33)	0.99	5.65 (1,33)	<0.05	0.23 ± 0.005	0.22 ± 0.004	4.5
M_sum_	36.8 (1,36)	<0.0001	16, 1 (1,36)	<0.001	—	—	1.65 ± 0.03	1.38 ± 0.03	19.6

**Table 3:** Correlations between BMR and body composition.

	Organ		Treatment		Interaction		Adjusted R^2^	BIC	∆BIC
	F (df)	P	F (df)	P	F (df)	P			
Total muscles^a^	9.8 (1,31)	<0.01	13.7 (1,31)	<0.001	—	—	0.33	−163.4	—
Skin	9.0 (1,31)	<0.01	11.9 (1,31)	<0.01	—	—	0.32	−162.7	−0.7
Carcass^a^	8.9 (1,31)	<0.01	14.6 (1,31)	<0.001	—	—	0.32	−162.6	−0.8
Legs^a^	6.4 (1,31)	<0.05	14.5 (1,31)	<0.001	—	—	0.28	−160.5	−3.0
Pectoralis	4.9 (1,31)	<0.05	5.1 (1,31)	<0.05	—	—	0.24	−159.0	−4.4
Lungs	1.8 (1,30)	0.19	8.0 (1,30)	<0.01	5.6 (1,30)	<0.05	0.26	−157.2	−6.2
Brain	2.6 (1,31)	0.11	9.1 (1,31)	<0.01	—	—	0.19	−156.8	−6.6
Kidneys	2.3 (1,31)	0.14	7.1 (1,31)	<0.05	—	—	0.19	−156.5	−6.9
Heart (wet)	0.99 (1,30)	0.33	6.1 (1,30)	<0.05	—	—	0.24	−156.3	−7.1
Intestine	6.1 (1,31)	<0.05	3.7 (1,30)	0.06	—	—	0.17	−155.6	−7.8
Gizzard	0.74 (1,31)	0.4	5.9 (1, 31)	<0.05	—	—	0.15	−154.9	−8.6
Pancreas	1.2 (1,28)	0.3	6.9 (1,28)	<0.05	—	—	0.15	−138.6	−24.8
Liver	0.12 (1,22)	0.7	2.1 (1, 21)	0.16	—	—	−0.04	−102.9	−60.6

Results are from final ANCOVA models, including lean dry mass of organ and treatment as variables.

^a^Includes bone mass (see text for details).

**Figure 2:**
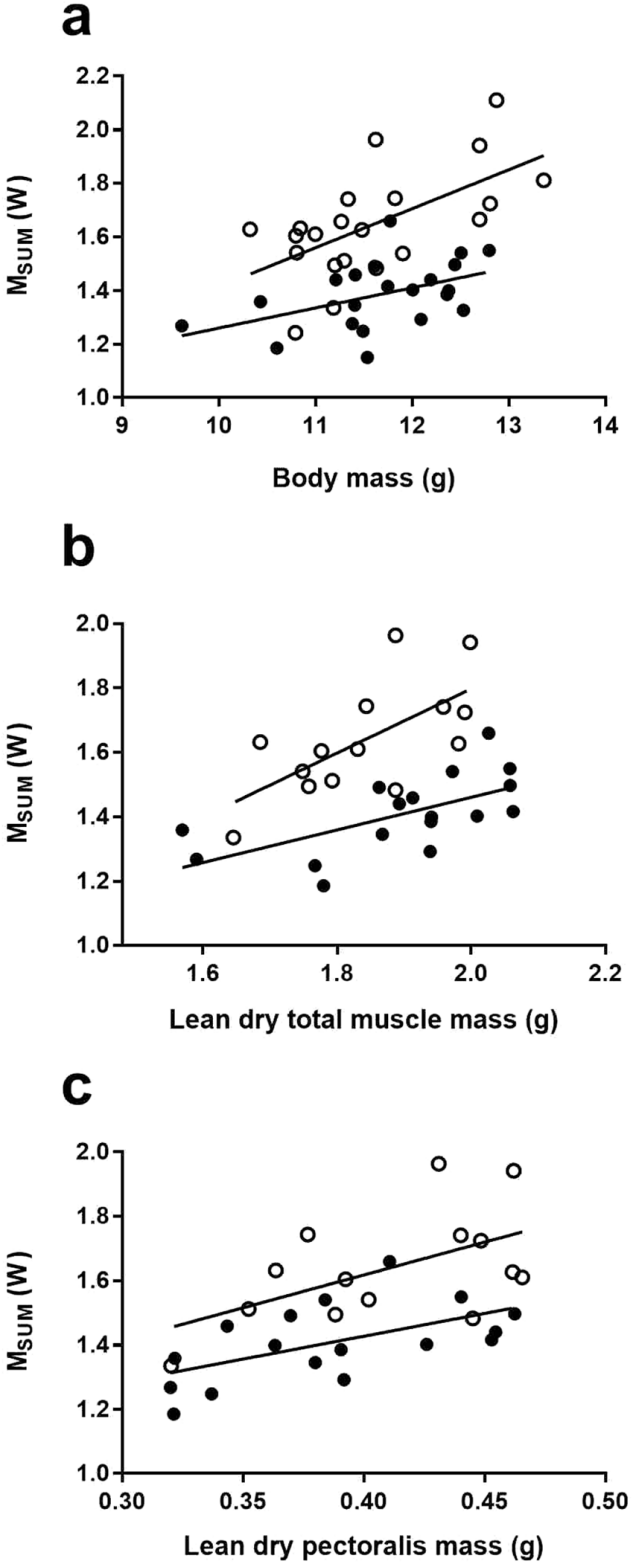
Relationships between M_sum_ and body mass and lean dry mass of skeletal muscles in black-capped chickadees: (**a**) body mass, (**b**) total lean dry muscle mass (including bones), (**c**) lean dry pectoralis muscle mass (filled circles: 27 °C, open circles: −10 °C).

M_sum_ was 19.6% higher in cold-acclimated birds when considering the significant effect of body mass (Table 2, Fig. 2a). As for BMR, muscles had a prominent influence on M_sum_ variation. Total muscles (Fig. 2b), pectoralis muscles (Fig. 2c), leg muscles and carcass were all positively correlated with M_sum_ and ranked first based on BIC values for independent ANCOVA models (Table 4).

**Table 4:** Correlations between M_sum_ and body composition.

	Organ		Treatment		Adjusted R^2^	BIC	∆BIC
	F (df)	P	F (df)	P			
Total muscles^a^	14.3 (1,25)	<0.001	31.7 (1,25)	<0.0001	0.33	−26.85	—
Pectoral	12.0 (1,25)	<0.01	13,9 (1,25)	<0.001	0.32	−25.2	−1.65
Legs^a^	10.7 (1,25)	<0.01	31.7 (1,25)	<0.0001	0.32	−24.1	−2.75
Carcass^a^	10.3 (1,25)	<0.01	29.1 (1,25)	<0.0001	0.28	−23.8	−3.05
Skin	4.2 (1,25)	0.05	20.5 (1,25)	<0.0001	0.24	−18.5	−8.35
Brain	3.8 (1,25)	0.06	20.5 (1,25)	<0.0001	0.26	−18.1	−8.75
Lungs	2.9 (1,25)	0.10	19.7 (1,25)	<0.001	0.19	−17.2	−9.65
Pancreas	1.7 (1,22)	0.21	19.6 (1,22)	<0.001	0.19	−15.9	−10.95
Gizzard	0.92 (1,25)	0.35	13.7 (1,25)	<0.01	0.24	−15.2	−11.65
Kidneys	0.1 (1,25)	0.75	15.0 (1,25)	<0.001	0.17	−14.3	−12.55
Heart (wet)	1.0 (1,24)	0.32	8.8 (1,24)	<0.01	0.15	−13.5	−13.35
Intestine	0.3 (1,24)	0.61	12.0 (1,24)	<0.01	0.15	−12.7	−14.15
Liver	0.2 (1,17)	0.65	15.1 (1,17)	<0.01	−0.04	−11.1	−15.75

Results are from final ANCOVA models, including lean dry mass of organ and treatment as variables. All interactions were non-significant.

^a^Includes bone mass (see text for details).

## Discussion

In this experiment, we expected higher thermogenic capacity in cold-acclimated birds relative to individuals kept at thermoneutrality and predicted that this difference would not result from larger skeletal muscles in the cold. Our data support this hypothesis as cold-acclimated individuals had a M_sum_ 20% higher but had smaller leg muscles and did not develop larger pectoralis, carcass or total muscles than birds kept at 27 °C. We also expected that variation in BMR would correlate with digestive and excretory organs if there were no major differences in muscle mass between treatments, or with skeletal muscles if their development was part of the response to cold. This hypothesis was only partially supported since skeletal muscles did correlate positively with BMR across treatment despite a lack of increase in muscle mass in the cold.

Birds acclimated to −10 °C consumed on average 44% more food per day during the last 6 days of acclimation than individuals kept at 27 °C. They also had 5% higher maintenance costs, based on average BMR, and 20% higher thermogenic capacity, based on average M_sum_. Therefore, the cold treatment was associated with considerable demands for thermoregulation and a consequent physiological response. Nevertheless, body mass did not differ between treatments. In fact, although we found the expected larger heart (based on wet mass) and larger pancreas in cold-acclimated birds, these individuals carried 27–30% less body fat and had smaller lungs and leg muscles than birds acclimated to thermoneutral conditions. The other components of lean body mass did not differ significantly between temperatures although most had higher mean values in cold acclimated birds (Table 1).

Similar body masses between contrasting thermal treatments have been reported before but previous captive experiments used milder cold exposure (e.g. 15 °C^38,61,62^). However, in a study using white throated sparrows, Barceló *et al*.^19^ reported that, when controlling for the effects of food consumption and body size, birds exposed to −8 °C had less body fat reserves, despite their heavier mass, compared to individuals kept at thermoneutrality (28 °C). Therefore, it appears, as suggested by Barceló *et al*.^19^, that the cold treatment experienced by our chickadees represented a considerable energy challenge as for a given amount of food consumed, less nutrients could be converted into fat reserves under cold conditions. Larger pancreases in the cold are likely attributable to higher food and protein intake^19,63,64^, but the exact causes for the smaller lungs and leg muscles are unclear. As birds maintained at −10 °C tended to be less active in their cage (M. Milbergue, unpublished observations), this could potentially be attributed to selective nutrient investment under a constrained energy budget where some organs like the heart and liver (+14% and +18% in the cold respectively) could be favored at the expanse of others.

The cold acclimated phenotype was associated with 5% higher maintenance costs on average but this effect differed between treatments when considering the influence of body mass. Indeed, we observed the typical relationship between BMR and body mass in birds at thermoneutrality but this relationship was uncoupled in cold-acclimated birds (Fig. 1a). This was not a simple effect of the range of data since both BMR and body mass spanned roughly the same range of values in both treatments (Fig. 1a). It was not a statistical “dilution” effect of body fat^53^ either since this effect lowers mass-corrected BMR values in fatter birds^53^, which in the present case were the birds kept at thermoneutrality. For this effect to take place, the lightest birds in the warm treatment (those with the lowest BMR) would have had to be the fattest, which was not the case (positive relationship between body and total organ fat mass at 27 °C, R^2^ = 0.26 n = 19 P < 0.05). Clearly, physiological changes in cold-acclimated birds has led to the observed pattern. The exact mechanism is unknown but, given the uncoupled effect of body mass on BMR, these changes are likely to have occurred at the cellular level^7,10,18,23,24,65–67^.

In the observed scenario where temperature did not influence skeletal muscle mass, we expected a significant effect of digestive and excretory organs on BMR^17,19^. Instead, total mass of muscles across treatments were positively related to maintenance costs. Despite the elevated food intake in the cold, none of the digestive and excretory organs, except pancreas, responded significantly to temperature and all of these organs ranked after skeletal muscles for their importance on explaining BMR. Skeletal muscles represent 73% of ash-free lean dry body mass in free-living wintering chickadees (Petit and Vézina, unpublished data) and pectoralis muscles alone represented 17% of total lean dry body mass in “our birds (Table 1)”. Therefore, without major changes in digestive and excretory organs, the energy consumed by resting skeletal muscles in birds under standard BMR conditions likely overshadowed the influence of other metabolically active organs such as the gut, liver or kidneys. This correlation between pectoralis or skeletal muscles and BMR has previously been observed in other avian species^25,62,68,69^, including free-living black capped chickadees^26^.

With regards to the relationship between skeletal muscle mass and BMR, an important point to consider is that it was only apparent in the thermoneutral group (see Fig. 1b,c). Independent regression models for total skeletal muscles, pectoralis, leg muscles and carcass were all significant at 27 °C (R^2^ = 0.35–0.54, all P < 0.01) but the same analyses for birds acclimated to −10 °C yielded no significant relationships (P > 0.6 in all cases). As stated earlier, and since most of lean body mass was made of skeletal muscles, this uncoupling in cold acclimated birds is likely resulting from changes in metabolic intensity taking place at the cellular level.

For a given body mass, cold-acclimated black capped chickadees had 20% higher thermogenic capacity than birds maintained at thermoneutrality (Fig. 2a). That difference did not result from larger skeletal muscles since these organs did not differ between groups (<1% difference in total muscle mass) or were smaller (legs) in cold-acclimated birds (Table 1). The combined mass of the heart and lungs has previously been shown to correlate with M_sum_ variation across seasons in our chickadee population^26^. Recent evidence also suggests that cardiovascular functions could play a significant role in thermogenic capacity as larger hearts^18,25,26,31,37,38,43,60,61^ and upregulated cardiac physiology^25,70,71^ are often found in association with cold acclimation or acclimatization in birds. In the current study, however, the lungs were smaller and the heart (based on wet mass) was larger in cold acclimated birds, but the mass of these organs did not correlate with M_sum_. Therefore, if cardiorespiratory function influences or limits thermogenic capacity^25^, its effect might result from system performance rather than from organ size.

Complementing previous observations in black capped chickadees^43^, dark-eyed juncos (*J. hyemalis*)^38,39^ and white-throated sparrows (*Z. albicollis*)^19^, our results support the hypothesis that the enlargement of skeletal muscles is not an absolute prerequisite for improving thermogenic capacity in small birds. Enhancement of maximal heat production in cold-acclimated chickadees could therefore result from changes occurring at the muscle cell level^19,39,71–73^ and these could take several forms. For example, Teulier *et al*.^72^ showed that muscovy ducklings (*Cairina moschata*) were able to increase heat production before the onset of leg muscles shivering at temperatures below the lower critical temperature (but note that this may not be the case in black-capped chickadees^74^). This was associated with an upregulation of avian uncoupling proteins (avUCP) in these same muscles, although the thermogenic role of avUCP could not be confirmed. Mathieu-Costello *et al*.^70^ further reported that king pigeons (*Colombia livia*) decreased aerobic muscle fiber diameter and increased muscle vascularization and mitochondrial volume density in response to cold acclimation. Similarly, Stager *et al*.^39^ observed an upregulation of pectoralis muscle genes known to play a role in muscle angiogenesis and repair in captive cold-acclimated juncos that had shown no increase in muscle mass.

Although M_sum_ was higher in cold acclimated birds for a given skeletal muscle mass (Fig. 2b,c), we also found, as predicted and previously observed in this and other species^10,26,32^ significant correlations between M_sum_ and lean dry mass of muscles in both cold and thermoneutral groups (Table 4, Fig. 2b,c). Consequently, while upregulating cell functions seems to be a requirement for improving thermogenic capacity in chickadees (see also^19^), our data also showed that birds with larger muscles still experienced the added benefit of generating more heat under acute cold stress and that this was independent from their acclimation temperature.

If larger muscles are not an absolute prerequisite for improving thermogenic capacity, then why are chickadees typically found with larger flight muscles in winter compared to summer [e.g. refs^26,31^]? One possibility is that winter locomotion for active foraging and daily fattening during cold, short working days requires a different flight pattern leading to larger flight muscles. Given that muscle mass correlates positively with M_sum_ at all temperatures, this hypothesis could also potentially explain why a number of individuals in our wild source population were found to maintain M_sum_ levels above that required to guarantee intra-winter survival^75^ if these individuals were also the most active in that population.

In sum, our experimental data showed a clear influence of muscle mass on both maintenance energy costs, measured as BMR, and maximal thermogenic capacity, measured as M_sum_, in black-caped chickadees. Our results also showed that, although large muscles may be beneficial in terms of heat production capacity, an increase in muscle size is clearly not required to elevate M_sum_ in these birds. Instead, improvement in thermogenic capacity appear to be related to cellular-level adjustments during cold-acclimation. The mechanisms underlying these adjustments deserves further study.

## Data Availability

The datasets generated and analysed during the current study are available from the corresponding author upon request.

## References

[1] Dawson WR, Carey C, Van’t Hof TJ (1992). Metabolic aspects of shivering thermogenesis in passerines during winter. Ornis Scand..

[2] Saarela S, Klapper B, Heldmaier G (1995). Daily rhythm of oxygen consumption and thermoregulatory responses in some European winter- or summer-acclimatized finches at different ambient temperatures. J. Comp. Physiol. B..

[3] O’Connor TP (1996). Geographic variation in metabolic seasonal acclimatization in house finches. Condor..

[4] Liknes ET, Swanson DL (1996). Seasonal variation in cold tolerance, basal metabolic rate, and maximal capacity for thermogenesis in white-breasted nuthatches *Sitta carolinensis* and downy woodpeckers. J. Avian Biol..

[5] Cooper SJ (2002). Seasonal metabolic acclimatization in mountain chickadees and juniper titmice. Physiol. Biochem. Zool..

[6] Swanson DL (1990). Seasonal variation in cold hardiness rates and peak rates of cold-induced thermogenesis in the dark-eyed junco (*Junco hyemalis*). The Auk..

[7] Zheng W-H, Li M, Liu J-S, Shao S-L (2008). Seasonal acclimatization of metabolism in Eurasian tree sparrows (*Passer montanus*). Comp. Biochem. Physiol. A Mol. Integr. Physiol..

[8] Petit M, Lewden A, Vézina F (2013). Intra-seasonal flexibility in avian metabolic performance highlights the uncoupling of basal metabolic rate and thermogenic capacity. PLoS One..

[9] McNab BK (1997). On the utility of uniformity in the definition of basal rate of metabolism. Physiol. Zool..

[10] McKechnie AE (2008). Phenotypic flexibility in basal metabolic rate and the changing view of avian physiological diversity: a review. J. Comp. Physiol. B..

[11] McKechnie AE, Swanson DL (2010). Sources and significance of variation in basal, summit and maximal metabolic rates in birds. Curr. Zool..

[12] Mckechnie AE, Noakes MJ, Smit B (2015). Global patterns of seasonal acclimatization in avian resting metabolic rates. J. Ornithol..

[13] Swanson, D. L., McKechnie, A. E. & Vézina, F. How low can you go? An adaptive energetic framework for interpreting basal metabolic rate variation in endotherms. *J. Comp. Physiol. B Biochem. Syst. Environ. Physiol*. 10.1007/s00360-017-1096-3 (2017). 10.1007/s00360-017-1096-3 28401293

[14] Swanson DL (2001). Are summit metabolism and thermogenic endurance correlated in winter-acclimatized passerine birds?. J. Comp. Physiol. B Biochem. Syst. Environ. Physiol..

[15] Swanson DL, Liknes ET (2006). A comparative analysis of thermogenic capacity and cold tolerance in small birds. J. Exp. Biol..

[16] Swanson DL (1991). Seasonal adjustments in metabolism and insulation in the dark-eyed junco. Condor..

[17] Williams JB, Tieleman BI (2000). Flexibility in basal metabolic rate and evaporative water loss among hoopoe larks exposed to different environmental temperatures. J. Exp. Biol..

[18] Zheng W-H (2013). Physiological and biochemical thermoregulatory responses of chinese bulbuls *Pycnonotus sinensis* to warm temperature: Phenotypic flexibility in a small passerine. J. Therm. Biol..

[19] Barceló, G., Love, O. P. & Vézina, F. Uncoupling basal and summit metabolic rates in white-throated sparrows: digestive demand drives maintenance costs, but changes in muscle mass are not needed to improve thermogenic capacity. *Physiol. Biochem. Zool*. **90**, 10.1086/689290 (2017). 10.1086/689290 28277963

[20] Piersma T (2002). Energetic bottlenecks and other design constraints in avian annual cycles. Integr. Comp. Biol..

[21] Vézina F, Gustowska A, Jalvingh KM, Chastel O, Piersma T (2009). Hormonal correlates and thermoregulatory consequences of molting on metabolic rate in a northerly wintering shorebird. Physiol. Biochem. Zool..

[22] Piersma T, Gessaman J, Dekinga A, Visser G (2004). Gizzard and other lean mass components increase, yet basal metabolic rated decrease, when red nots *Calidris canutus* are shifted from soft to hard-shelled food. J. Avian Biol..

[23] Vézina F, Williams TD (2005). Interaction between organ mass and citrate synthase activity as an indicator of tissue maximal oxidative capacity in breeding European Starlings: implications for metabolic rate and organ mass relationships. Funct. Ecol..

[24] Swanson DL (2010). Seasonal metabolic variation in birds: functional and mechanistic correlates. In Current Ornithology..

[25] Vézina F, Gerson AR, Guglielmo CG, Piersma T (2017). The performing animal: causes and consequences of body remodeling and metabolic adjustments in red knots facing contrasting thermal environments. Am. J. Physiol.-Regul. Integr. Comp. Physiol..

[26] Petit M, Lewden A, Vezina F (2014). How does flexibility in body composition relate to seasonal changes in metabolic performance in a small passerine wintering at northern latitude?. Physiol. Biochem. Zool..

[27] Hohtola E, Stevens ED (1986). The relationship of muscle electrical activity, tremor and heat production to shivering thermogenesis in Japanese quail. J. Exp. Biol..

[28] Saarela S, Heldmaier G (1987). Effect of photoperiod and melatonin on cold resistance, thermoregulation and shivering/non-shivering thermogenesis in Japanese quail. J. Comp. Physiol. B..

[29] Swanson DL (1991). Substrate metabolism under cold stress in seasonally acclimatized dark-eyed juncos. Physiol. Zool..

[30] O’Connor TP (1995). Metabolic characteristics and body composition in house finches: effects of seasonal acclimatization. J. Comp. Physiol. B..

[31] Liknes ET, Swanson DL (2011). Phenotypic flexibility of body composition associated with seasonal acclimatization in passerine birds. J. Therm. Biol..

[32] Swanson DL, Vézina F (2015). Environmental, ecological and mechanistic drivers of avian seasonal metabolic flexibility in response to cold winters. J. Ornithol..

[33] Swanson DL, King MO, Culver W, Zhang Y (2017). Within-winter flexibility in muscle masses, myostatin, and cellular aerobic metabolic intensity in passerine birds. Physiol. Biochem. Zool..

[34] Vézina F, Jalvingh KM, Dekinga A, Piersma T (2006). Acclimation to different thermal conditions in a northerly wintering shorebird is driven by body mass-related changes in organ size. J. Exp. Biol..

[35] Vézina F, Dekinga A, Piersma T (2011). Shorebirds’ seasonal adjustments in thermogenic capacity are reflected by changes in body mass: How preprogrammed and instantaneous acclimation work together. Integr. Comp. Biol..

[36] Swanson DL, Zhang Y, King MO (2013). Individual variation in thermogenic capacity is correlated with flight muscle size but not cellular metabolic capacity in american goldfinches (*Spinus tristis*). Physiol. Biochem. Zool..

[37] Swanson D, Zhang Y, King M (2014). Mechanistic drivers of flexibility in summit metabolic rates of small birds. PLoS One..

[38] Swanson DL, Zhang Y, Liu J-S, Merkord CL, King MO (2014). Relative roles of temperature and photoperiod as drivers of metabolic flexibility in dark-eyed juncos. J. Exp. Biol..

[39] Stager M, Swanson DL, Cheviron ZA (2015). Regulatory mechanisms of metabolic flexibility in the dark-eyed junco (*Junco hyemalis*). J. Exp. Biol..

[40] Hohtola, E. Shivering thermogenesis in birds and mammals. In *Life in the cold: evolution, mechanisms, adaptation, and application. Twelfth International Hibernation Symposium* (ed. Barnes B. M., Carey, H. V.), 241–252, Fairbanks: Institute of Arctic Biology, University of Alaska (2004).

[41] Cooper SJ, Swanson DL (1994). Seasonal acclimatization of thermoregulation in the black-capped chickadee. Condor..

[42] Petit M, Vezina F (2014). Phenotype manipulations confirm the role of pectoral muscles and haematocrit in avian maximal thermogenic capacity. J. Exp. Biol..

[43] Swanson DL, King MO, Harmon E (2014). Seasonal variation in pectoralis muscle and heart myostatin and tolloid-like proteinases in small birds: A regulatory role for seasonal phenotypic flexibility?. J. Comp. Physiol. B Biochem. Syst. Environ. Physiol..

[44] Rising JD, Hudson JW (1974). Seasonal variation in the metabolism and thyroid activity of the black-capped chickadee (*Parus atricapillus*). Condor..

[45] Lewden A, Petit M, Vézina F (2012). Dominant black-capped chickadees pay no maintenance energy costs for their wintering status and are not better at enduring cold than subordinate individuals. J. Comp. Physiol. B Biochem. Syst. Environ. Physiol..

[46] Swanson DL, Drymalski MW, Brown JR (1996). Sliding vs static cold exposure and the measurement of summit metabolism in birds. J. Therm. Biol..

[47] Prinzinger R, Preßmar A, Schleucher E (1991). Body temperature in birds-mini review. Comp. Biochem. Physiol.–Part. A Mol. Integr. Physiol..

[48] Cooper SJ, Gessaman JA (2005). Nocturnal hypothermia in seasonally acclimatized mountain chickadees and juniper titmice. Condor..

[49] Lighton, J. R. B. *Measuring metabolic rate: a manual for scientists*. Oxford, UK: Oxford University Press (2008).

[50] Vaillancourt E, Prud’homme S, Haman F, Guglielmo CG, Weber J-M (2005). Energetics of a long-distance migrant shorebird (*Philomachus pugnax*) during cold exposure and running. J. Exp. Biol..

[51] Gessaman AJ, Nagy KA (1988). Energy metabolism: errors in gas-exchange conversion factors. Physiol. Zool..

[52] Scott I, Evans PR (1992). The metabolic output of avian (*Sturnus vulgaris, Calidris alpina*) adipose tissue liver and skeletal muscle: Implications for BMR/body mass relationships. Comp. Biochem. Physiol. Comp. Physiol..

[53] Petit M, Vézina F, Piersma T (2010). Ambient temperature does not affect fuelling rate in absence of digestive constraints in long-distance migrant shorebird fuelling up in captivity. J. Comp. Physiol. B Biochem. Syst. Environ. Physiol..

[54] Gosler A (1996). Environmental and social determinants of winter fat storage in the great tit. Parus major. J. Anim. Ecol..

[55] Rising JD, Somers KM (1989). The measurement of overall body size in birds. The Auk..

[56] Piersma T, Davidson NC (1991). Confusion of mass and size. The Auk..

[57] Senar JC, Pascual J (1997). Keel and tarsus may provide a good predictor of avian body size. Ardea..

[58] Nespolo RF, Bacigalupe LD, Sabat P, Bozinovic F (2002). Interplay among energy metabolism, organ mass and digestive enzyme activity in the mouse-opossum *Thylamys elegans*: the role of thermal acclimation. J. Exp. Biol..

[59] Quinn, G. P. & Keough, M. J. *Experimental design and data analysis for biologists*. Cambridge, UK: Cambridge University Press (2002).

[60] Zheng W-H, Liu J-S, Swanson DL (2014). Seasonal phenotypic flexibility of body mass, organ masses, and tissue oxidative capacity and their relationship to resting metabolic rate in chinese bulbuls. Physiol. Biochem. Zool..

[61] Maldonado KE, Cavieres G, Veloso C, Canals M, Sabat P (2009). Physiological responses in rufous-collared sparrows to thermal acclimation and seasonal acclimatization. J. Comp. Physiol. B..

[62] Peña-Villalobos I, Nuñez-Villegas M, Bozinovic F, Sabat P (2014). Metabolic enzymes in seasonally acclimatized and cold acclimated rufous-collared sparrow inhabiting a Chilean Mediterranean environment. Curr. Zool..

[63] Karasov WH, Pinshow B, Starck JM, Afik D (2004). Anatomical and histological changes in the alimentary tract of migrating blackcaps (*Sylvia atricapilla*): a comparison among fed, fasted, food‐restricted, and refed Birds. Physiol. Biochem. Zool..

[64] Fassbinder-Orth CA, Karasov WH (2006). Effects of feed restriction and realimentation on digestive and immune function in the Leghorn chick. Poult. Sci..

[65] Rolfe DFS, Brown GC (1997). Cellular energy utilization and molecular origin of standard metabolic rate in mammals. Physiol. Rev..

[66] Liu J-S, Chen Y-Q, Li M (2006). Thyroid hormones increase liver and muscle thermogenic capacity in the little buntings (*Emberiza pusilla*). J. Therm. Biol..

[67] Zheng W-H, Lin L, Liu J-S, Xu X-J, Li M (2013). Geographic variation in basal thermogenesis in little buntings: Relationship to cellular thermogenesis and thyroid hormone concentrations. Comp. Biochem. Physiol.- A Mol. Integr. Physiol..

[68] Chappell MA, Bech C, Buttemer WA (1999). The relationship of central and peripheral organ masses to aerobic performance variation in house sparrows. J. Exp. Biol..

[69] Vézina F, Williams TD (2003). Plasticity in body composition in breeding birds: what drives the metabolic costs of egg production?. Physiol. Biochem. Zool..

[70] Mathieu-Costello O (1998). Fiber capillarization and ultrastructure of pigeon pectoralis muscle after cold acclimation. J. Exp. Biol..

[71] Zhang Y, Eyster K, Liu J-S, Swanson DL (2015). Cross-training in birds: cold and exercise training produce similar changes in maximal metabolic output, muscle masses and myostatin expression in house sparrows, Passer domesticus. J. Exp. Biol..

[72] Teulier L (2010). Cold-acclimation-induced non-shivering thermogenesis in birds is associated with upregulation of avian UCP but not with innate uncoupling or altered ATP efficiency. J. Exp. Biol..

[73] Liknes ET, Swanson DL (2011). Phenotypic flexibility in passerine birds: Seasonal variation of aerobic enzyme activities in skeletal muscle. J. Therm. Biol..

[74] Chaplin SB (1976). The physiology of hypothermia in the Black-capped chickadee, Parus atricapillus. J. Comp. Physiol..

[75] Petit M, Clavijo-Baquet S, Vézina F (2017). Increasing winter maximal metabolic rate improves intrawinter survival in small birds. Physiol. Biochem. Zool..

